# Effects of stabilized hypochlorous acid on oral biofilm bacteria

**DOI:** 10.1186/s12903-022-02453-2

**Published:** 2022-09-20

**Authors:** Olivia Aherne, Roberto Ortiz, Magnus M. Fazli, Julia R. Davies

**Affiliations:** 1 Section for Oral Biology and Pathology, Faculty of Odontology and Biofilms Research Center for Biointerfaces, Malmö University, 205 06 Malmö, Sweden; 2 CR Competence, Naturvetarvägen 14, 223 62 Lund, Sweden; 3 Costerton Biofilm Center, Department of Immunology and Microbiology, University of Copenhagen, Copenhagen, Denmark; 4 SoftOx Solutions AS, Copenhagen, Denmark

**Keywords:** Biofilm control, Oral disease, Caries, Periodontitis, Oral infection

## Abstract

**Background:**

Caries and periodontitis are amongst the most prevalent diseases worldwide, leading to pain and loss of oral function for those affected. Prevention relies heavily on mechanical removal of dental plaque biofilms but for populations where this is not achievable, alternative plaque control methods are required. With concerns over undesirable side-effects and potential bacterial resistance due to the use of chlorhexidine gluconate (CHX), new antimicrobial substances for oral use are greatly needed. Here we have investigated the antimicrobial effect of hypochlorous acid (HOCl), stabilized with acetic acid (HAc), on oral biofilms and compared it to that of CHX. Possible adverse effects of stabilized HOCl on hydroxyapatite surfaces were also examined.

**Methods:**

Single- and mixed-species biofilms of six common oral bacteria (*Streptococcus mutans*, *Streptococcus gordonii*, *Actinomyces odontolyticus*, *Veillonella parvula*, *Parvimonas micra* and *Porphyromonas gingivalis*) within a flow-cell model were exposed to HOCl stabilized with 0.14% or 2% HAc, pH 4.6, as well as HOCl or HAc alone. Biofilm viability was assessed in situ using confocal laser scanning microscopy following LIVE/DEAD® BacLight™ staining. *In-situ* quartz crystal microbalance with dissipation (QCM-D) was used to study erosion of hydroxyapatite (HA) surfaces by stabilized HOCl.

**Results:**

Low concentrations of HOCl (5 ppm), stabilized with 0.14% or 2% HAc, significantly reduced viability in multi-species biofilms representing supra- and sub-gingival oral communities, after 5 min, without causing erosion of HA surfaces. No equivalent antimicrobial effect was seen for CHX. Gram-positive and Gram-negative bacteria showed no significant differential suceptibility to stabilized HOCl.

**Conclusions:**

At low concentrations and with exposure times which could be achieved through oral rinsing, HOCl stabilized with HAc had a robust antimicrobial activity on oral biofilms, without causing erosion of HA surfaces or affecting viability of oral keratinocytes. This substance thus appears to offer potential for prevention and/or treatment of oral biofilm-mediated diseases.

**Supplementary Information:**

The online version contains supplementary material available at 10.1186/s12903-022-02453-2.

## Introduction

Comprised of hard and soft tissue surfaces, the human oral cavity provides an array of niches that facilitate the development of a highly complex microbiome, with over 600 different species so far identified [1, 2]. High diversity in bacterial colonization is enhanced by the presence of a pellicle on oral surfaces, which is mainly composed of proteins adsorbed from salivary secretions. Receptors within this protein film act as binding sites for surface adhesin-expressing bacteria, so-called early colonizers, with bacterial attachment providing a range of new binding sites for later colonizers [3, 4]. This sequential accumulation of bacteria over time gives rise to oral biofilms (dental plaque), with the heaviest microbial load on non-shedding tooth surfaces. When in balance with the host (eubiosis), multi-species biofilms on tooth and mucosal surfaces represent a critical factor for oral health, by impeding colonization of pathogenic species [5]. However, as elucidated by the ecological plaque hypothesis, environmental perturbation such as changes in nutrient levels can affect both the composition and phenotype of these normally protective biofilms, giving rise to dysbiosis [6]. Affecting more than 3,5 billion people worldwide [7], the oral diseases; caries and periodontitis, are both associated with dysbiosis in oral biofilms. In addition to causing disease in the oral cavity, dysbiotic plaque biofilms are increasingly recognized as potential reservoirs for pathogens that can establish infections at other sites [8, 9].

Currently, the best method available for caries and periodontitis prevention is the mechanical removal of biofilms from tooth surfaces. However, this strategy relies heavily on patient compliance and may not be feasible for certain population groups such as those with physical or intellectual disabilities. Hence, the use of supplementary plaque control methods may be warranted. Chlorhexidine gluconate (CHX) is one of the most common antimicrobial substances used in the oral cavity and has potent effects on the oral microbiome [10]. A recent systematic review concluded that there was high-quality evidence that use of a CHX mouthwash as an adjuvant therapy to mechanical removal procedures leads to large reductions in dental plaque burden [11]. However, experimental studies have shown that, due to its high molecular weight and charge, penetration of CHX into established biofilms in the absence of mechanical disruption is poor, killing only superficial bacteria while leaving bacteria in the deeper layers unaffected [12, 13]. Moreover, prolonged use of CHX is associated not only with dental pigmentation, mucosal injury and mucosal drying [14] but also, as highlighted in a recent review, development of resistance both to CHX and cross-resistance to antibiotics [15].

Hypochlorous acid (HOCl) is an endogenous antimicrobial substance of the innate immune response, produced as part of the respiratory burst during bacterial killing by neutrophils and macrophages [16]. HOCl (often in its anionic form, OCl^−^) has long been used as an antibacterial substance ex vivo due to its powerful oxidative effect, which leads to multiple effects on macromolecules and membrane-related processes in microorganisms including oxidation of intracellular proteins, inhibition of DNA synthesis, oxidation of membrane lipids and inhibition of ATP synthesis [17, 18]. In the light of the increasing global threat of antimicrobial resistance, interest has grown in using HOCl to treat biofilm-mediated human infections in vivo. However, a potential challenge in developing HOCl as an antiseptic product is the maintenance of stability during storage, as HOCl tends to dissociate into its component ions over time [19]. This issue can be resolved by the addition of low concentrations of an acetic acid (HAc) buffer, which stabilizes the pH, thereby maintaining an optimum concentration of HOCl within the solution. Since HAc is also recognized as an antimicrobial substance [20], it may also act as an ancillary agent to the HOCl solution. Several recent studies have suggested that stabilized HOCl may be a suitable agent for the treatment of bacterial infections in acute and chronic wounds [21, 22]. Due to its low molecular weight that allows rapid diffusion, and lack of charge that prevents it being repelled from negatively charged bacterial cell surfaces, HOCl has potential for use as an adjunctive antimicrobial substance in the oral cavity. A recent clinical trial observed a good antimicrobial effect of HOCl on bacteria in saliva but no experiments on sessile bacteria were conducted [23]. As oral bacteria predominantly exist as surface-associated biofilms, our aim was to investigate the antimicrobial effect of HOCl, stabilized with two different concentrations of HAc, against a range of oral bacteria grown in single and mixed-species biofilms and to compare the effect with that of CHX. An important property of any product used in the oral cavity is that it should not cause erosion of the tooth surface, therefore we have also studied the effect of stabilized HOCl on hydroxyapatite (HA) surfaces as a model for tooth enamel.

## Materials and methods

### Bacterial strains and culture media

The following archived bacterial strains were used throughout this study: *Streptococcus gordonii* (JD13A) from healthy oral mucosa, *Streptococcus mutans* (B4B), *Veillonella parvula* (10BB), *Actinomyces odontolyticus* (G3H) and *Actinomyces naeslundii* [110BT (catalase positive) and CW (catalase-negative)], all originally from dental plaque of a healthy subject; *Parvimonas micra* (EME), originally from the sub-gingival pocket of a patient with peri-implant disease and the type strain of *Porphyromonas gingivalis* (W50). Identities of the strains had been confirmed by 16S *r*RNA gene sequencing. All strains were stored in skim milk (Oxoid) at − 80 °C. *Streptococci* were grown on blood agar overnight in 5% CO_2_ in air at 37 °C, whereas all other species were routinely cultured on blood or Brucella agar (*P. gingivalis*) for up to 7 days at 37 °C under anaerobic conditions (10% H_2_, 5% CO_2_ in N_2_). Multi-species biofilms were grown overnight in protein-rich nutrient medium (PRNM) developed by Naginyte et al. [24] without the porcine gastric mucin.

### Preparation of antimicrobial substances

Acetic acid (HAc) solutions (0.125%, 0.25%, 0.5%, 1%, 2%, 6%) were prepared through dilution of glacial acetic acid (Sigma-Aldrich) in distilled water. Solutions were buffered to pH 2.3 or 4.6 using NaOH and/or HCl and kept at room temperature. Solutions of hypochlorous acid (HOCl) (0.5 ppm, 1 ppm, 5 ppm, 10 ppm, 20 ppm, 100 ppm, corresponding to 9.53 µM-1.9 mM HOCl) were made by dissolving sodium hypochlorite pentahydrate (Tokyo Chemical Industry) in distilled water. Solutions were buffered to pH 4.6 using perchloric acid (Sigma-Aldrich) and stored in the dark at 4 °C for up to 3 weeks. Confirmation of initial concentrations was performed using spectrophotometry at A_236_ and comparison against a standard curve. Stabilized HOCl solutions (0.5 ppm, 1 ppm, 5 ppm, 10 ppm, 20 ppm) in HAc buffer were made on the day of use from a HOCl stock solution (100 ppm) (provided by SoftOx Solutions AB) containing set concentrations of HAc (0.14% or 2%), buffered to pH 4.6. Stock solutions stored in the dark at 4 °C were shown to be stable (< 0.04% reduction in concentration of HOCl) for at least 3 months under these conditions. Chlorhexidine (CHX) solutions [0.5 ppm, 1 ppm, 5 ppm, 10 ppm, 20 ppm, 100 ppm (corresponding to 0.99 µM–0.198 mM)] were prepared by dilution in autoclaved distilled water from a stock solution of 20% chlorhexidine gluconate in water (Sigma-Aldrich, St Louis, MI, USA).

### Effects of antimicrobial substances on single- and multi-species biofilms

Suspensions (OD_600_ = 0.1) of individual bacterial species were prepared in 25% Todd-Hewitt (TH) broth (BD Biosciences, NJ, USA). For *P. micra*, the 25% TH broth was supplemented with 500 µg/mL L-cysteine. Bacterial suspensions were then added to channels in Ibidi® µ-slide VI Ibi-treat flow-cells (Ibidi GmbH, Gräfelfing, Germany) and incubated in a humid chamber under anaerobic/CO_2_ conditions at 37 °C for 16–24 h to allow biofilm formation on the slide surface.

For biofilms representing supra-gingival (*S. mutans*, *A. odontolyticus* and *V. parvula*) and sub-gingival (*S. gordonii*, *P. gingivalis* and *P. micra*) communities, bacterial species were mixed in approximately equal amounts in phosphate-buffered saline (PBS; 0.15 M NaCl, 0.05 M NaH_2_PO_4_, pH 7.4). Suspensions (200 µL) were added to channels in the Ibidi® flow-cells and incubated in a humid chamber under anaerobic conditions for 4 h to allow for bacterial attachment. Following this, the PBS was exchanged for PRNM to allow bacterial growth, and the flow-cells were  then incubated for 24 h. All broths and PBS were pre-reduced to anaerobic conditions prior to experimentation. Biofilms were incubated with test solutions at room temperature for 5 min and then slides were rinsed twice with PBS.

### Vital fluorescence microscopy and image analysis

Following exposure to the test or control solutions, biofilms were stained in situ for 15 min in the dark using LIVE/DEAD® BacLight™ viability stain (Molecular Probes, Eugene, OR, USA) containing Syto 9 (green) and propidium iodide (red). Syto 9 enters all cells but in those with a compromised membrane, the green staining is quenched out by the entry of propidium iodide turning the dead cells red. Stained biofilms were viewed using a Nikon Eclipse TE200 inverted confocal laser scanning microscope. Image analysis to determine the total biofilm surface coverage and percentage of viable (green) cells was performed using the colour segmentation software *bio*Image_L [25] on 10 randomly selected images from each experimental condition. All experiments were undertaken in triplicate using independent biological replicates. Test conditions were compared with the control (25% TH broth) using the Kruskal Wallis test in GraphPad Prism with Dunns' post-test for mutliple comparisons. *p* values < 0.05 were considered statistically significant.

### Effect of stabilized HOCl on viability of oral keratinocytes

An MTT assay (Abcam Inc.) was used to determine the effect of stabilized HOCl solutions on the viability of oral keratinocytes. Immortalized human oral keratinocytes (OKF6/TERT-2, p33) were seeded into 96-well tissue culture plates and grown to confluency in keratinocyte serum-free medium (k-sfm) from Thermo Fisher Scientific, Paisley, UK, supplemented with 0.2 ng/mL human recombinant EGF, 25 µg/mL bovine pituitary extract and 0.3 mM CaCl_2_ containing 1 IU/mL penicillin and 1 µg/mL streptomycin. Cells were treated with the test solutions (1, 5, 10 and 20 ppm HOCl stabilized with 0.14% in k-sfm) for 10 min. Following exposure, solutions were replaced with a 1:1 ratio of MTT reagent in PBS, and incubated for 3 h at 37 °C to allow formazan crystal formation. The MTT reagent was then carefully removed, replaced with MTT solvent and placed on a shaker. Concentrations of formazan were then determined through absorbance at 590 nm. Test conditions were compared with the control (k-sfm) using the Kruskal Wallis test in GraphPad Prism with Dunns' post-test for mutliple comparisons. *p* values < 0.05 were considered statistically significant.

### *In-situ* quartz crystal microbalance with dissipation (in-situ QCM-D)

**Fig. 1:**
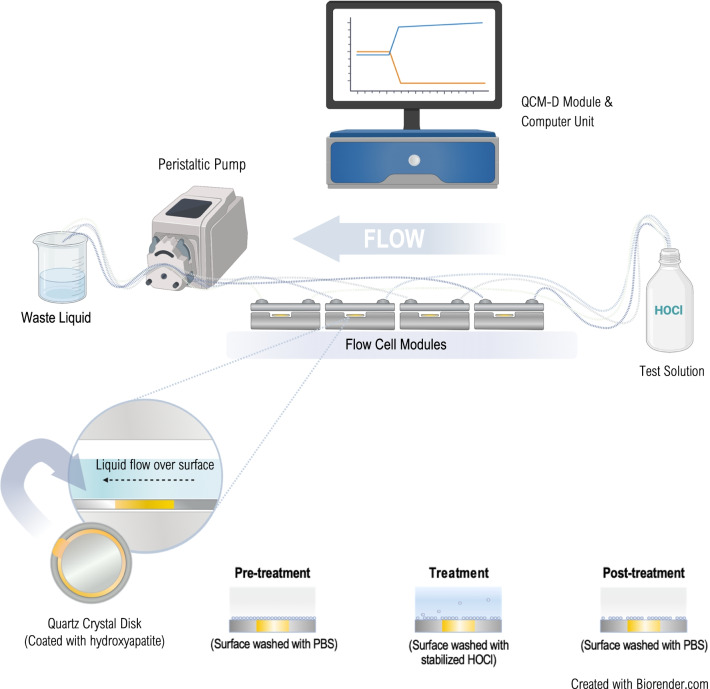
A schematic image of the set-up used in the in-situ quartz crystal microbalance with dissipation (in-situ QCM-D) experiments. Briefly, clean QCM-D sensor chips were mounted on the instrument and PBS flushed through the system until a stable baseline of ∆*f* and ∆*D* was observed. Test solutions were then flowed over the hydroxyapatite surface for 20 min, followed by a PBS rinse for 15 min

Measurement of hydroxyapatite (HA) surface erosion was performed using a Q-sense E4 system (Biolin Scientific AB, Gothenburg, Sweden) equipped with a peristaltic pump from Ismatec (Wertheim, Germany) (Fig. 1). The QCM-D method is based on the change in resonant frequency of a vibrating quartz crystal sensor, a piezoelectric material, according to mass changes of the sensor. The QCM-D instrument monitors real-time changes in the frequency of vibrational modes as well as changes in the vibrational energy dissipation. Chamber temperature was maintained at 22.0 °C ± 0.02 °C and a flow rate of 100 µL/min was used for all the experiments. The AT-cut piezoelectric HA-coated quartz crystal disks used as the sensor chip (Q-sense, Biolin Scientific AB, Gothenburg, Sweden) had a fundamental frequency of 4.95 MHz ± 50 kHz and vibrate in the thickness-shear mode with the overtone n of 1, 3, 5, 7, 9, 11 and 13. Before use, sensor chips were UV-treated for 20 min, then immersed in 99% ethanol for 30 min, rinsed extensively with milli-Q water and blow-dried using nitrogen gas. Finally, the QCM-D sensors were UV-treated for 20 min. Clean QCM-D sensor chips were mounted directly on the QCM-D instrument and used immediately. All solutions were degassed by sonication prior to use. First, ∆*f* was measured in air to evaluate the correct mounting of the sensor chip and ethanol was then introduced in the cell to eliminate possible air bubbles over the surface. PBS was subsequently flushed through the system until a stable baseline of ∆*f* and ∆*D* was observed. Test solutions (1, 5, 10 and 20 ppm HOCl stabilized with 0.14% HAc or 2% HAc) in addition to 0.14% and 2% HAc alone and positive controls; 15% HAc solution (pH 2.3) and 5% HCl (pH 0.3) were flowed over the HA surface for 20 min, followed by a PBS rinse for 15 min. All experiments were performed in duplicate. The data presented correspond to the change in vibrational frequency and its associated dissipation (∆*f*n = ∆*F*n/n and ∆*D*) of the sensor chip vs. time. The measured frequency shift for the fifth overtone (∆*f*_5_) was used to evaluate the effect of the solutions on the HA surfaces.

## Results

### Effect of stabilized HOCl on multi-species biofilms

**Fig. 2:**
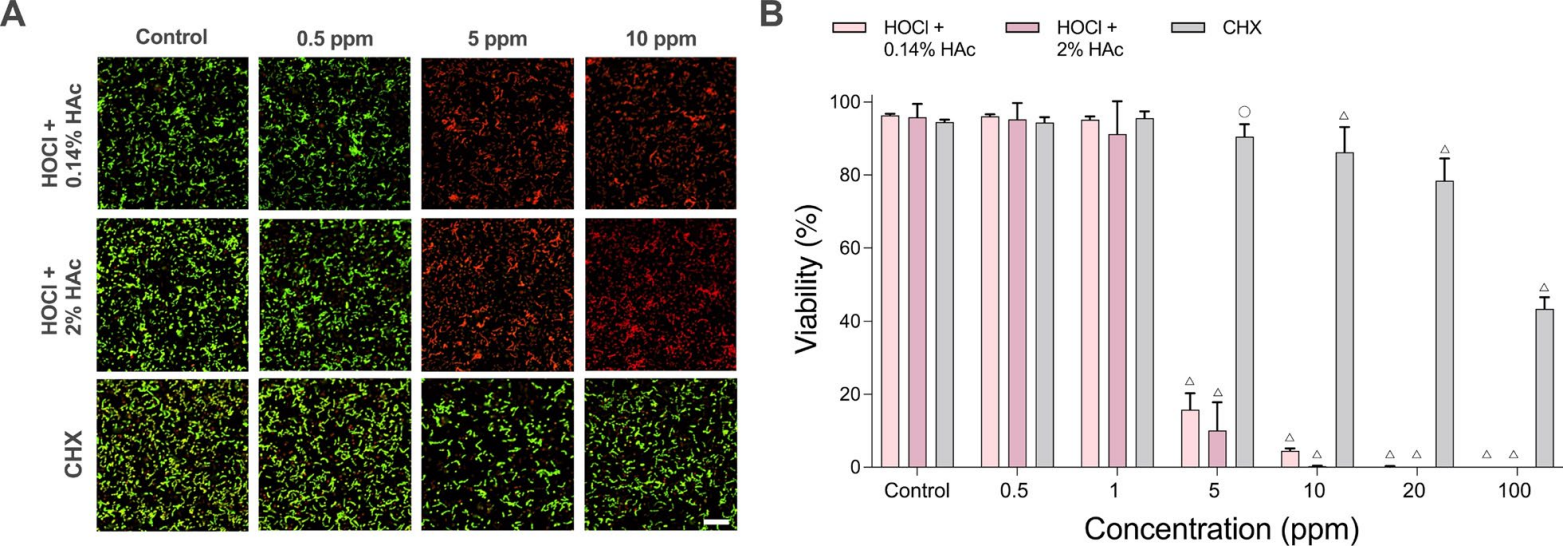
Effect of stabilized HOCl and CHX on multi-species biofilms modelling supra-gingival communities. **A** Representative images showing biofilms treated for 5 min with 25% TH broth (control), increasing concentrations of HOCl (stabilized with 0.14% or 2% HAc) or CHX, and stained with LIVE/DEAD® BacLight™ viability stain. The scale bar shows 10 µm. **B** Bar charts showing mean viability ± SD obtained by analysis of 10 random images from each of three independent biological replicates (open circle = *p* ≤ 0.05, open triangle = *p* ≤ 0.0001)

Model biofilms representing supra- and sub-gingival oral communities, were created to investigate the effects of treatment with HOCl solution stabilized with two different concentrations of HAc on the coverage and viability of multi-species biofilms. For the supra-gingival biofilm, viability was significantly reduced after treatment for 5 min with 5 ppm stabilized HOCl compared to control (Fig. 2A, B). At this concentration, buffering the HOCl solution with 2% HAc appeared to have a greater effect on viability than 0.14% HAc, but this difference was not significant. At 10 ppm stabilized HOCl almost no bacteria survived. In contrast, treatment with equivalent concentrations of CHX had little effect, and even at concentrations as high as 100 ppm CHX, viability within the biofilm remained approximately 40%. Surface coverage in the biofilms treated with stabilized HOCl or CHX showed only minor differences from control suggesting that, although the bacteria are killed, the biofilms are not detached to any great degree by either treatment (data not shown).

**Fig. 3:**
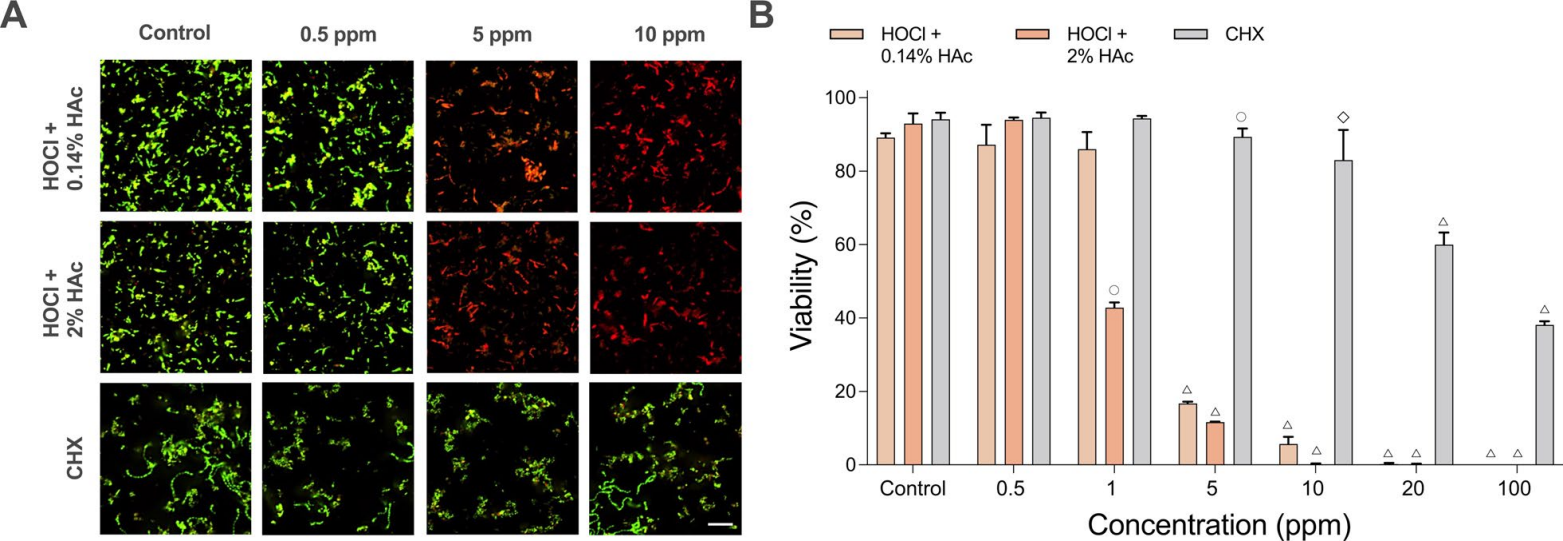
Effect of stabilized HOCl and CHX on multi-species biofilms modelling sub-gingival communities. **A** Representative images showing biofilms treated for 5 min with 25% TH broth (control), increasing concentrations of HOCl (stabilized with 0.14% or 2% HAc) or CHX, and stained with LIVE/DEAD® BacLight™ viability stain. The scale bar shows 10 µm. **B** Bar charts showing mean viability ± SD obtained by analysis of 10 random images from each of three independent experiments (open circle = *p* ≤ 0.05, open diamond = *p* ≤ 0.001, open triangle = *p* ≤ 0.0001)

The sub-gingival community showed a similar pattern to that of the supra-gingival biofilms for 0.14% HAc, with viability significantly reduced with 5 ppm stabilized HOCl and almost no survival at 10 ppm (Fig. 3A, B). However, biofilms treated with HOCl buffered with 2% HAc showed a significant reduction (approximately 50%) in viability at HOCl concentrations as low as 1 ppm. Similar to the supragingival biofilm, 40% biofilm viablility was observed for sub-gingival biofilms following treatment with 100 ppm CHX for 5 min. No significant removal of the sub-gingival biofilms was seen after treatment for 5 min with either the stabilized HOCl or CHX solutions (data not shown).

### Effect of stabilized HOCl on individual biofilm species

**Fig. 4:**
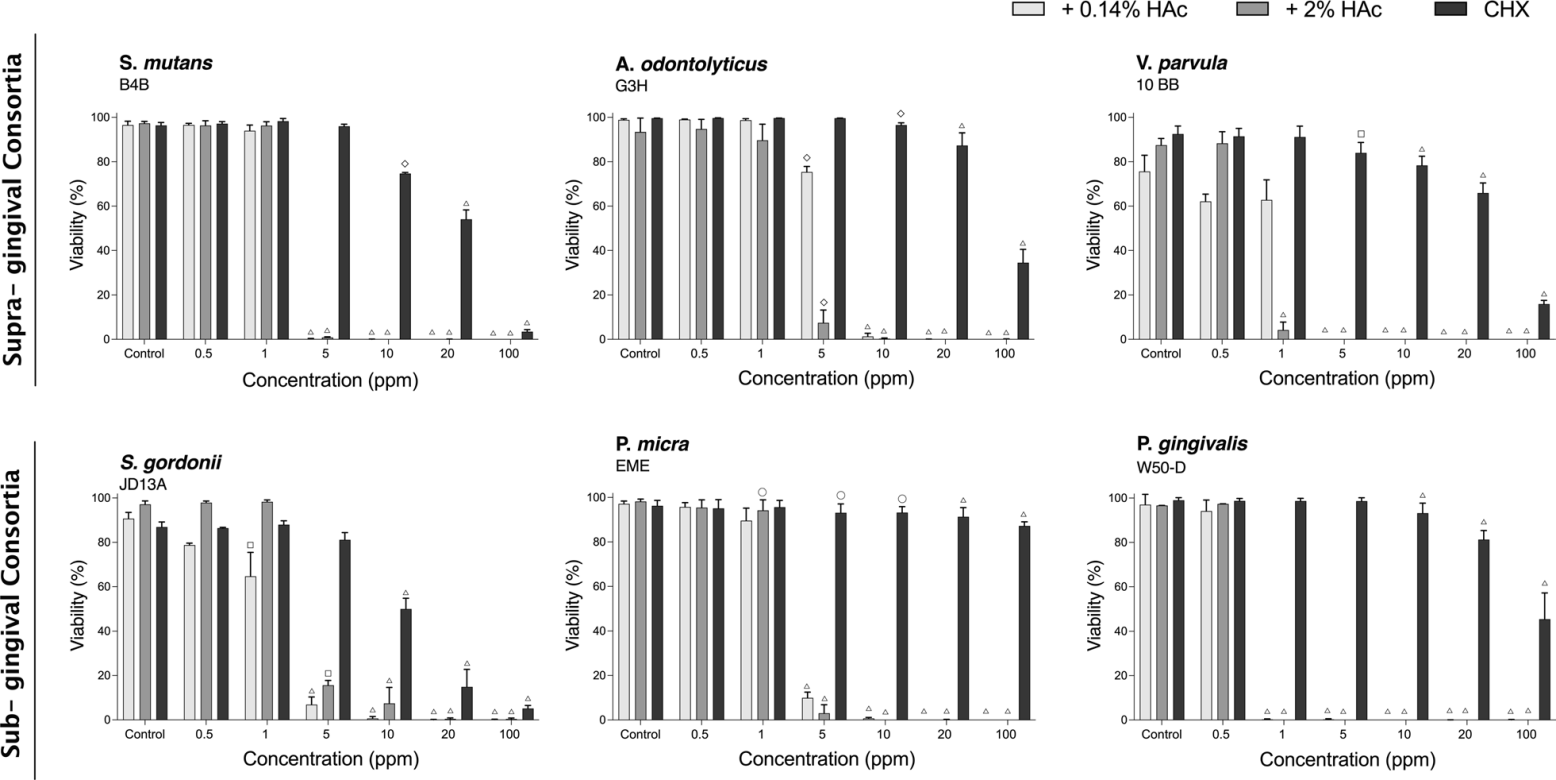
Effect of treatment with stabilized HOCl and CHX on single-species biofilms of six oral bacteria. Bar charts showing mean viability ± SD (% green cells after staining with LIVE/DEAD® BacLight™ viability stain) obtained by analysis of 10 random images from each of three independent experiments for biofilms treated for 5 min with 25% TH broth (control), increasing concentrations of HOCl (stabilized with 0.14% or 2% HAc) or CHX (open circle = *p* ≤ 0.05, open square = *p* ≤ 0.01, open  diamond = *p* ≤ 0.001, open triangle = *p* ≤ 0.0001)

To investigate whether stabilized HOCl exerted a differential bactericidal action on the individual species comprising the multi-species biofilms, as well as to determine whether Gram-positve and Gram-negative bacteria were affected differently, the effect of short-term treatment with stabilized HOCl was also carried out on mono-species biofilms. For biofilms of Gram-positive cocci, *S. mutans* and Gram-negative cocci, *V. parvula,* exposure to 5 ppm HOCl for 5 min caused complete killing of the biofilm bacteria, irrespective of the HAc buffer concentration (Fig. 4). For *V. parvula*, HOCl buffered with 2% HAc also showed a significant killing effect at 1 ppm. In contrast, the Gram-positive bacillus *A. odontolyticus* was more resistant; with approximately 80% and 10% survival at 5 ppm HOCl in 0.14% HAc and 2% HAc respectively (Fig. 4). These findings thus suggest that the bacteria surviving within the supra-gingival biofilm at 5 ppm HOCl most likely represent mainly *A. odontolyticus*.

The effect of stabilized HOCl also varied between species within the sub-gingival biofilm. In biofilms of Gram-positive *S. gordonii* and Gram-negative *P. micra*, survival levels at 1 ppm stabilized HOCl were high, but a sharp decline in viability was noted on exposure to 5 ppm HOCl for 5 min, irrespective of the HAc buffer concentration (Fig. 4). The most sensitive bacteria was the Gram-negative species; *P. gingivalis*, where no survival was seen at concentrations as low as 1 ppm HOCl (Fig. 4). Taken together, these data show differences in the sensitivity to stabilized HOCl between the bacterial strains tested, which do not appear to be directly related to their cell envelope characteristics. Consistent with the results seen for the multi-species biofilms, low concentrations of CHX were much less effective than stabilized HOCl at killing sessile bacteria, although the effects on individual species varied. *Streptococcus* species showed a gradual reduction in biofilm viability as the CHX concentration increased from 5 to 100 ppm, while *A. odontolyticus*, *P. gingivalis* and *P. micra* biofilms still showed significant levels of viability at 100 ppm (Fig. 4).

### Effect of HAc and HOCl on single-species biofilms

**Fig. 5:**
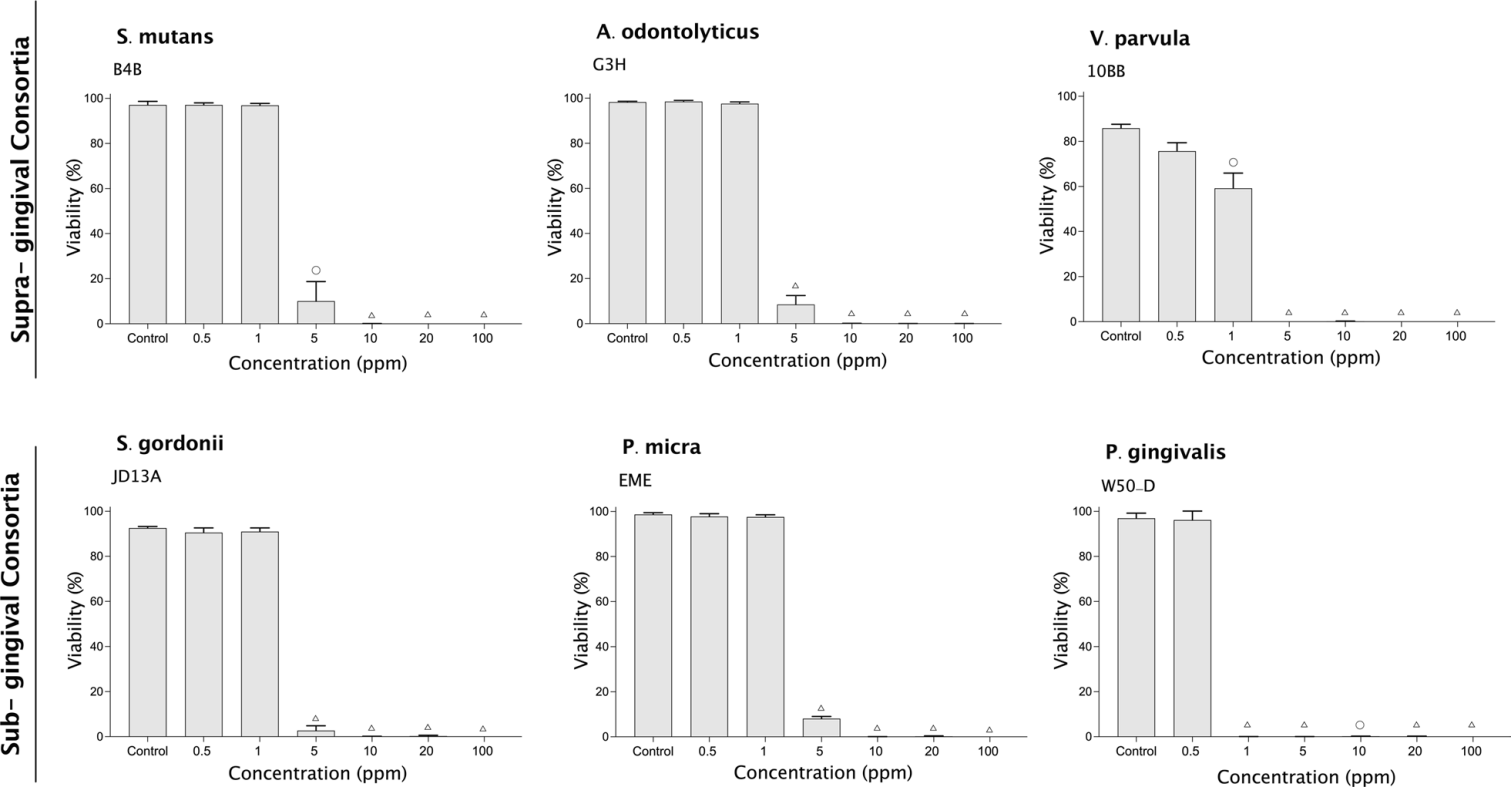
Effect of treatment with HOCl on single-species biofilms of six oral bacteria. Bar charts showing mean viability ± SD (% green cells after staining with LIVE/DEAD® BacLight™ viability stain) obtained by analysis of 10 random images from each of three independent experiments for biofilms treated for 5 min with 25% TH broth (control) or increasing concentrations of HOCl (open circle = *p* ≤ 0.05, open square = *p* ≤ 0.01, open triangle = *p* ≤ 0.0001)

Since both HAc and HOCl have been demonstrated to have antimicrobial properties individually, each was tested separately on the single-species biofilms to examine their individual contributions to the bactericidal effects observed for the stabilized HOCl solution. At pH 4.6, concentrations of HAc between 0.125% and 6% showed no effect on viability of these oral bacteria (Additional file 1: Fig. S1). However at pH 2.3, HAc had a markedly different effect depending on the species tested. *Streptococcus mutans* showed high sensitivity, with almost complete killing after exposure to 0.125% HAc for 5 min while *S. gordonii* and *V. parvula* showed some reduction in viability at concentrations between 0.125 and 6% HAc. However, *A. odontolyticus*, *P. micra* and *P. gingivalis* showed high levels of viability even after exposure to concentrations as high as 6% HAc (Additional file 1: Fig. S1). Biofilm exposure to HOCl alone at pH 4.6 closely mirrored the effects seen for stabilized HOCl solution, with all species except *P. gingivalis* showing high levels of viability at 1 ppm and a significant reduction in survival at 5 ppm (Fig. 5). These data thus suggest that the effect of stabilized HOCl solution is largely due to the HOCl component, although at low concentrations, the presence of 2% HAc appeared to enhance the antimicrobial effect against some of the species tested.

### Influence of catalase production on susceptibility to HOCl

**Fig. 6:**
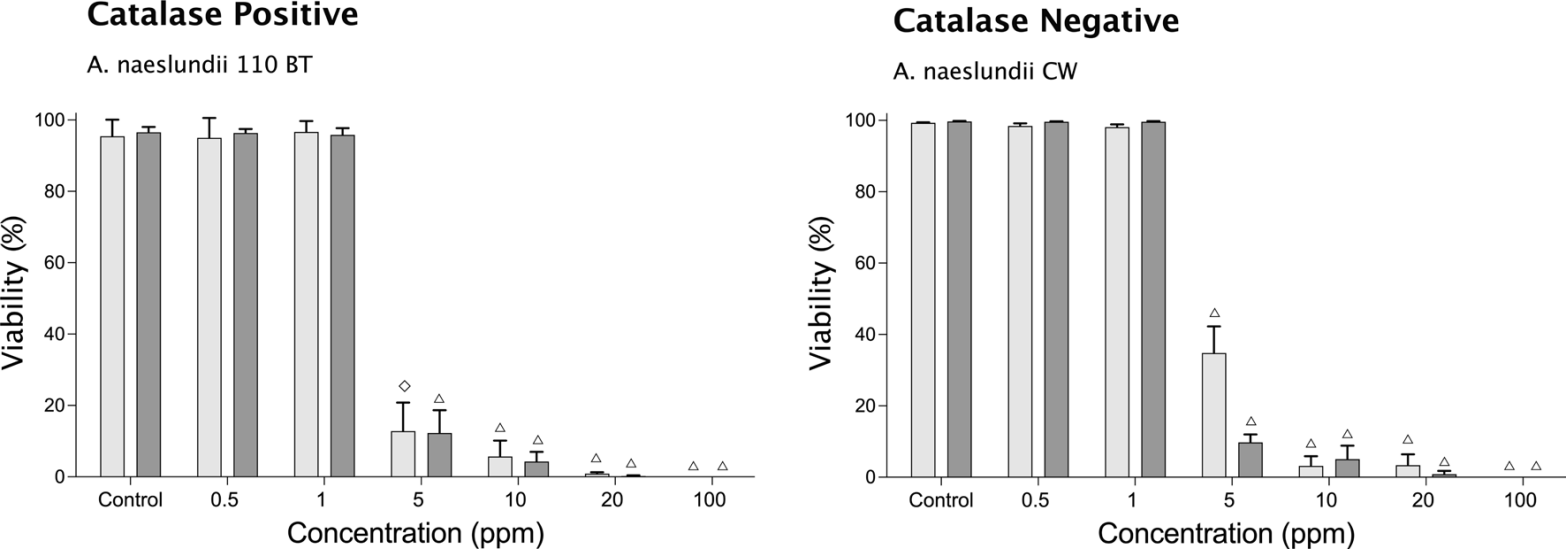
Effect of treatment with stabilized HOCl on single-species biofilms of catalase-positive and catalase-negative strains of *A. naeslundii*. Bar charts showing mean viability ± SD (% green cells after staining with LIVE/DEAD® BacLight™ viability stain) obtained by analysis of 10 random images from each of three independent experiments for biofilms treated for 5 min with 25% TH broth (control) or increasing concentrations of stabilized HOCl (open  diamond = *p* ≤ 0.001, open triangle = *p* ≤ 0.0001)

Previous work has suggested that the ability of bacteria to produce catalase may confer resistance to oxidative agents such as HOCl through quenching of the oxidative effect independent of the catalytic effect. Therefore, in this study, the effect of stabilized HOCl was tested on two strains of *Actinomyces naeslundii*; one catalase positive (110BT) and one catalase negative (CW). No significant differences in suceptibility between the strains were seen, suggesting that the ability to produce catalase was not protective under these conditions (Fig. 6).

### Effect of stabilized HOCl on oral keratinocytes and hydroxyapatite surfaces

**Fig. 7:**
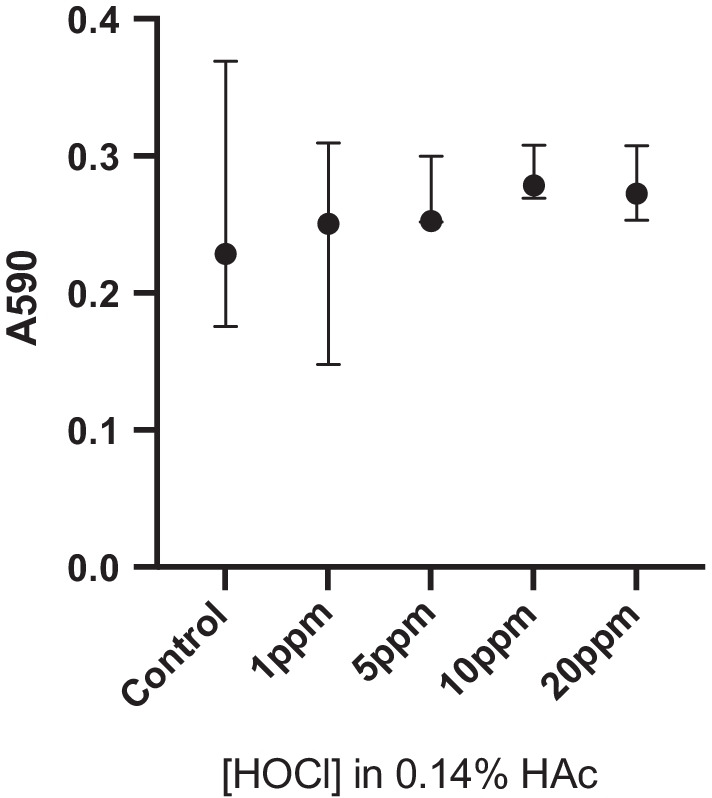
Effect of stabilized HOCl on oral keratinocytes. Cell viability after exposure for 10 min to 1, 5, 10 or 20 ppm HOCl in 0.14% HAc was examined using an MTT assay. The graph shows the median and range for absorbance at 590 nm from three independent replicates. Analysis was carried out using a Kruskal Wallis test with Dunn's test for multiple comparisons

**Fig. 8:**
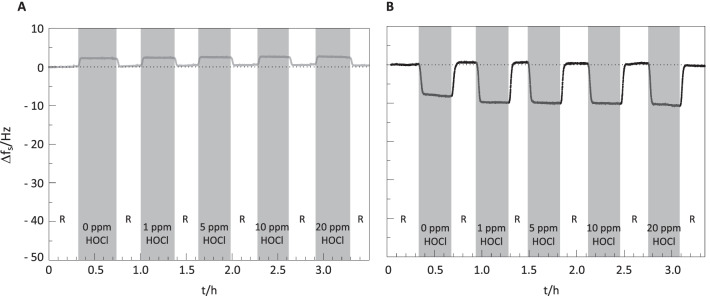
QCM-D data showing the shift of Δ*f*_5_ values for 1–20 ppm HOCl stabilized with **A** 0.14% or **B** 2% HAc as a function of exposure time. The final concentration of HOCl is indicated inside each grey bar zone. Grey and white zones indicate contact between the HA surface and test solutions or rinsing with Milli Q water respectively

The effect of stabilized HOCl on oral keratinocytes was examined using an MTT assay. This showed that, compared to control, concentrations of up to 20 ppm HOCl for 10 min had no significant cytotoxic effect (Fig. 7). The potential of stabilized HOCl to cause erosion from the tooth surface was studied using in situ QCM-D. Exposure of the HA coated sensor chips to increasing concentrations of HOCl in 0.14% or 2% HAc for 20 min caused shifts in the measured frequency for the fifth overtone (∆*f*_5_) of approximately 2 Hz and − 10 Hz, respectively (Fig. 8A, B). After rinsing with PBS for 15 min, Δ*f*_*5*_ returned to the pre-treatment value (0 Hz). Thus, the values of Δ*f*_*5*_ remain essentially constant after each treatment with the stabilized HOCl, with small variations between concentrations lying within the sensitivity limit of the instrument. Changes in Δ*f*_*5*_ observed when the 0.14% HAc and 2% HAc solutions were in contact with the sensors were due to the differences in the bulk effect compared to the PBS [26]. Together these results indicate that none of the tested solutions (1, 5, 10 and 20 ppm HOCl stabilized with 0.14% HAc or 2% HAc), or 0.14% and 2% HAc alone, caused erosion detectable with QCM-D. When HA surfaces were exposed to 15% HAc or 5% HCl, the Δ*f*_*5*_ varied to initial values of -47 and − 12 Hz, respectively (Additional file 2: Fig. S2). During the treatment with the acids, Δ*f*_*5*_ exhibited a drift to more positive values. This effect was more pronounced for HCl solution and is interpreted as erosion of the surface (i.e. removal of HA). After rinsing with physiological saline solution the values of Δ*f*_*5*_ varied to approximately 1 Hz for the 15% HAc solution and to 3.5 Hz for the 5% HCl solution, indicating that Δ*f*_*5*_ did not return to the original value of 0 Hz. This can be interpreted as a loss of mass occurring at the HA surface, consistent with erosion of the HA from the QCM-D sensor.

## Discussion

Caries and periodontitis are amongst the most prevalent of all human diseases, estimated to affect around 621 and 743 million people worldwide [26, 27]. Mechanical removal of plaque from the tooth surfaces is currently the most widely used intervention for the prevention of these biofilm-induced conditions. However, this approach requires a high level of compliance and alternative or adjunctive therapy may be needed for some patient groups. To investigate the suitability of the endogenous antimicrobial substance, HOCl, for use in the oral cavity, we have explored the effects of low concentrations of HOCl on the viability of oral bacteria. In this study, treatment of the biofilms was carried out for 5 min to model the approximate exposure levels obtained during mouth rinsing. Since biofilm bacteria are known to be more tolerant to antimicrobial agents than their planktonic counterparts [28], a mini-flow cell system, where the antimicrobial effects could be examined in situ*,* combined with viability staining was used as a model system. In the supra-gingival biofilm model, prepared using bacterial species commonly found in initial caries lesions [29], treatment for 5 min with a low concentration of HOCl (10 ppm) caused complete loss of viability, irrespective of the concentration of HAc buffer (0.14 or 2%) used. Similar results were obtained for the biofilm modelling the simplified sub-gingival commmunity [30] although in 2% HAc, significant loss of viability was obtained at concentrations of HOCl as low as 1 ppm. This suggest that at low concentrations of HOCl, the antimicrobial effect against some bacterial species may be enhanced by the presence of 2% HAc. This effect was superior to that seen for CHX, at concentrations of up to 100 ppm. Thus, even at low concentrations, HOCl stabilized with either 0.14% or 2% HAc has a robust antimicrobial effect against multi-species oral biofilms, using exposure times comparable to those that could be obtained during rinsing with an oral product. These results correlate well with clinical investigations showing that products containing NaOCl can reduce plaque index and gingival bleeding in vivo [31–33] although in this investigation, no significant detachment of the biofilms was seen.

Both the supra- and sub-gingival biofilms were prepared using a mixture of Gram-positive and Gram-negative species. Since previous studies have shown CHX to have a greater effect on Gram-positive than Gram-negative bacteria [34], the effect of stabilized HOCl was tested on each of the component species individually to assess whether there were differences in their suceptibility that could be linked to differences in the structure of the cell envelope. Data showed that the Gram-positive species; *A. odontolyticus* had a somewhat lower sensitvity to HOCl than Gram-negative *P. gingivalis*, with the effect on other species falling in between. Differences were small and consistent with a previous study of planktonic cultures of Gram-positive and Gram-negative oral bacteria showing that both were equally sensitive to low levels of HOCl in weakly acidic solution [35]. Some oral bacteria have the ability to produce catalase, an enzyme known to play a major role in resistance to reactive-oxygen species [36] and upregulated in bacteria such as *Bacillus subtilis* [37] and *P. aeruginosa* [38] in response to reactive chlorine species. Therefore to examine whether catalase-production would afford a degree of protection against the anti-microbial effects of HOCl, two strains of *A. naeslundii*, one catalase-positive and the other catalase-negative were examined. The results showed no differences in susceptibility suggesting that, in this system, catalase production conferred no protection against the anti-microbial action of HOCl. Thus, overall, the absence of differential resistance between the tested species suggests that treatment of oral biofilms with low concentrations of HOCl would not lead to significant shifts in biofilm composition over time.

Acetic acid used to stabilize the HOCl solution [39], has also been shown to have antimicrobial properties against both oral and skin bacteria growing in biofilms [20]. Therefore, the possibility of an ancilliary effect of HAc in the mixture was investigated by treating the single-species biofilms separately with HAc or HOCl. Treatment revealed no effect of HAc at pH 4.6 on biofilm viability of any of the species tested, whereas HOCl reduced viability at similar concentrations to those observed using the stabilized mixture. Thus the antibacterial effect of stabilized HOCl against the oral bacteria tested here appears to be mainly due to HOCl. This is supported by the lack of significant differences in effect between HOCl stabilized with 0.14% HAc and that stabilized with 2% HAc. At pH 2.3 however, the *streptococci* tested showed reduced viability after exposure to HAc for 5 min, while *A. odontolyticus*, *V. parvula*, *P. micra* and *P. gingivalis* were largely resistant. In a previous study, biofilms of the skin colonizers *Pseudomonas aeruginosa* and *Staphylococcus aureus*, were completely eradicated by treatment with HAc at pH 4.35 for 24 h [20]. The high levels of resistance seen in this study are not fully understood but may be related to the time of exposure and/or the presence of adaptive mechanisms in oral bacteria due to frequent exposure to HAc as a dietary component and/or end-point metabolite in oral biofilms [40].

A pH value of 4.6 was chosen for the stabilized HOCl in order to maintain optimum levels of HOCl in its non-ionized form and thus maximize uptake into bacterial cells. As this is lower than the critical pH value for tooth enamel, the effect of exposure of HA to HOCl stabilized with 0.14% or 2% HAc was investigated using QCM-D. This technique, which has been used employed previously in erosion studies [41, 42], detects changes in mass on surfaces down to the ng/cm^2^ level [43], thus allowing for evaluation of surface erosion at the nanoscale level. The results obtained showed no changes in response to short-term exposure to the stabilized HOCl solutions indicating that they did not cause erosion of the HA surface. Investigation of the effects on keratinocyte viability also showed that stabilized HOCl was not cytotoxic at concentrations that had an antimicriobial effect.

In summary, this study shows that at low concentrations and with short exposure times, HOCl stabilized with HAc has a robust antimicrobial activity against biofilms of a range of different oral bacteria, without causing erosion of HA surfaces or affecting keratinocyte viability. In the light of concerns regarding development of resistance to antibiotics and even CHX, this substance appears to offer potential for the prevention and treatment of oral biofilm-mediated diseases. Further studies are now required to investigate the efficacy of stabilized HOCl in vivo, where factors such as substantivity and the role of saliva can be assessed.

## Supplementary Information

**Additional file 1.** Effect of treatment with HAc on single-species biofilms of six oral bacteria. Bar charts showing mean viability ± SD (% green cells after staining with LIVE/DEAD® BacLightTM viability stain) obtained by analysis of 10 random images from each of three independent experiments for biofilms treated for 5 minutes with 25% TH broth (control) or increasing concentrations of HAc (open square = *p* ≤ 0.01, open triangle = *p* ≤ 0.0001).

**Additional file 2.** QCM-D data showing the shift of Δf5 values for solutions of 15% HAc (red zone, pH 2.3) and 5% HCl (green zone, pH 0.3) as a function of exposure time. White zones (R) show rinsing of the HA surface with Milli Q water.

## Data Availability

All datasets used and/or analysed during the current study are available from the corresponding author on reasonable request.
