# Supplementary material for: Effects of stabilized hypochlorous acid on oral biofilm bacteria
Source: BMC Oral Health. 2022 Sep 20;22:415. doi: 10.1186/s12903-022-02453-2 (PMC9487106; doi:10.1186/s12903-022-02453-2)
Supplement: Supplementary file 1 — Additional file 1. Effect of treatment with HAc on single-species biofilms of six oral bacteria. Bar charts showing mean viability ± SD (% green cells after staining with LIVE/DEAD® BacLightTM viability stain) obtained by analysis of 10 random images from each of three independent experiments for biofilms treated for 5 minutes with 25% TH broth (control) or increasing concentrations of HAc (open square = p ≤ 0.01, open triangle = p ≤ 0.0001). [file 12903_2022_2453_MOESM1_ESM.pdf]

Supplementary Fig. 1

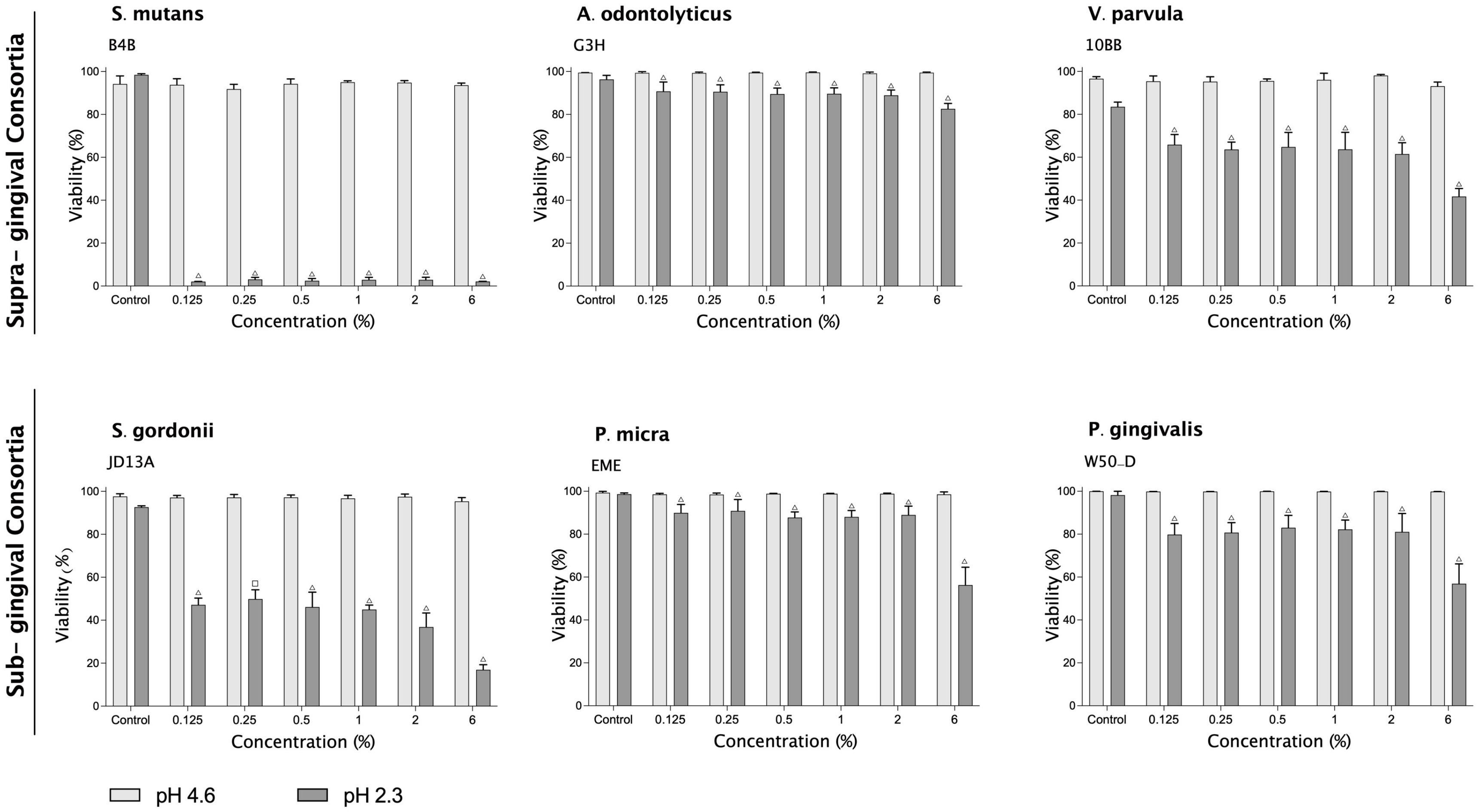

Supplementary Fig. 1. Effect of treatment with HAc on single-species biofilms of six oral bacteria. Bar charts showing mean viability ± SD (% green cells after staining with LIVE/DEAD® BacLight[TM] viability stain) obtained by analysis of 10 random images from each of three independent experiments for biofilms treated for 5 minutes with 25% TH broth (control) or increasing concentrations of HAc (o = $p \leq 0.05$, ☐ = $p \leq 0.01$, ◇ = $p \leq 0.001$, Δ = $p \leq 0.0001$).
