# Supplementary material for: Effects of stabilized hypochlorous acid on oral biofilm bacteria
Source: BMC Oral Health. 2022 Sep 20;22:415. doi: 10.1186/s12903-022-02453-2 (PMC9487106; doi:10.1186/s12903-022-02453-2)
Supplement: Supplementary file 2 — Additional file 2. QCM-D data showing the shift of Δf5 values for solutions of 15% HAc (red zone, pH 2.3) and 5% HCl (green zone, pH 0.3) as a function of exposure time. White zones (R) show rinsing of the HA surface with Milli Q water. [file 12903_2022_2453_MOESM2_ESM.pdf]

Supplementary Fig. 2

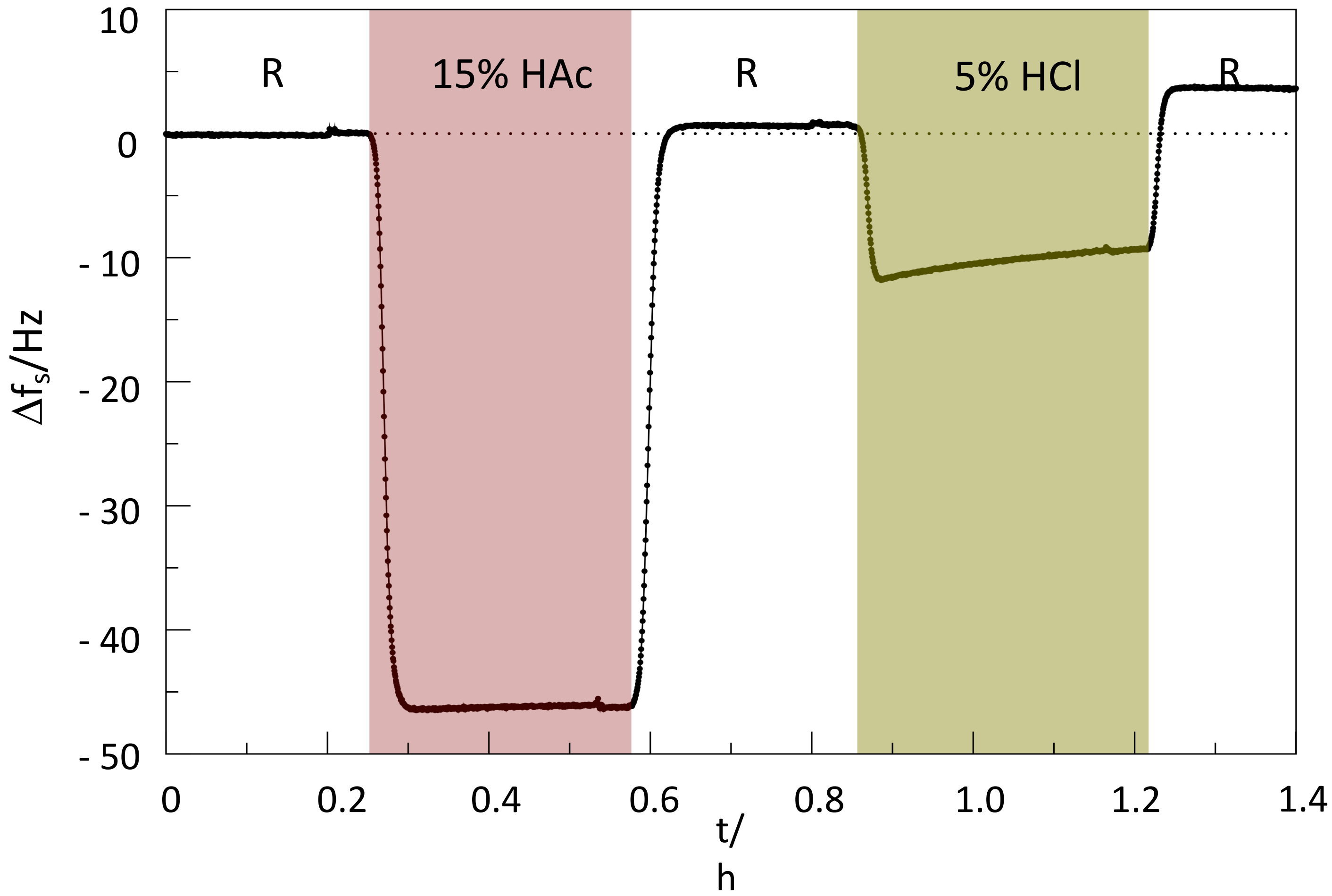

Supplementary Fig. 2. QCM-D data showing the shift of Δf_5 values for solutions of 15% HAc (red zone, pH 2.3) and 5% HCl (green zone, pH 0.3) as a function of exposure time. White zones (R) show rinsing of the HA surface with Milli Q water.
